# Zur Erinnerung an Prof. Dr. med. Manfred Strietzel

**DOI:** 10.1007/s00066-022-02018-w

**Published:** 2022-11-04

**Authors:** Thomas Herrmann, Johannes Schorcht, Guido Hildebrandt

**Affiliations:** 1 Medizinische Fakultät Carl Gustav Carus, Technische Universität Dresden, Dresden, Deutschland; 2 Klinik für Strahlentherapie, Univ.-Medizin Rostock, Rostock, Deutschland

**Figure 1:**
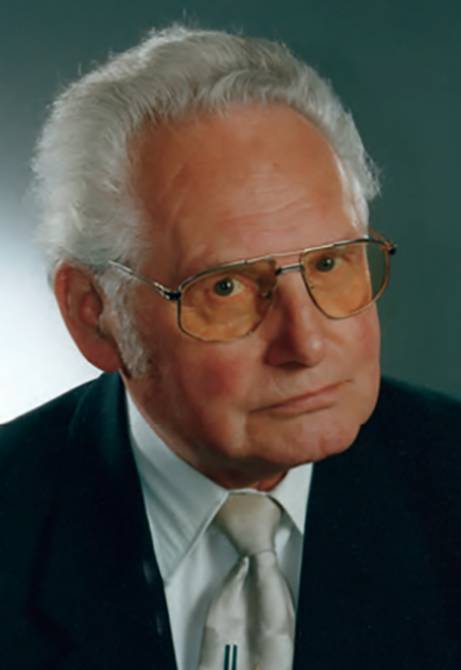

Prof. Manfred Strietzel (Abb. 1) ist am 6. September im Alter von 94 Jahren in Rostock verstorben. Damit verliert die ostdeutsche Radioonkologie den letzten Nestor unseres Fachs, dessen berufliche Expertise von der Einführung der Telekobalttherapie 1957 bis zur Entwicklung leistungsfähiger Linearbeschleuniger und von der LDR-Brachytherapie bis zur modernen Afterloading-Therapie reichte.

Manfred Strietzel wurde in Kleinschönau/Niederschlesien geboren, legte das Abitur in Zittau ab und studierte Humanmedizin in Leipzig. Nach einer internistischen Ausbildung im Bergmanns-Krankenhaus Klettwitz begann er 1956 seine Ausbildung in der Radiologie und war ab 1959 an der Medizinischen Akademie Dresden in diesem Fach tätig. Nach seinem zweiten Facharztabschluss in Radiologie wechselte er dauerhaft in die Strahlentherapie und baute eine der größten ostdeutschen radioonkologischen Abteilungen in Dresden auf, die er als Abteilungsleiter bis 1978 leitete.

In diesem Jahr wurde er anfangs zum Hochschuldozenten und 1980 zum Professor für Strahlentherapie mit eigener großer Klinik an die medizinische Fakultät der Universität Rostock berufen. Seine Tätigkeit in Rostock wurde durch die Eintragung ins Ehrenbuch der Stadt 1979 und mit dem Universitätspreis 1986 gewürdigt. Ab 1991 war Prof. Strietzel geschäftsführender Direktor der Radiologischen Klinik und Poliklinik und wurde 1992 gemäß § 3 Hochschulerneuerungsgesetz zum Universitätsprofessor (C4) Radiologie/Strahlentherapie an der Universität Rostock ernannt. Diese Funktion übte er bis zu seiner Emeritierung im Jahr 1996 aus. Prof. Strietzel war maßgeblich an der Gründung des Tumorzentrums Rostock im Jahr 1991 beteiligt und war bis 1998 Vorsitzender dieses neu gegründeten interdisziplinären Zentrums. Im gleichen Zeitraum (1990–1999) leitete er die Fachkommission Strahlentherapie an der Ärztekammer Mecklenburg-Vorpommern.

Sowohl in Dresden als auch in Rostock plante und realisierte er Neubauten für das Fach Strahlentherapie, die in ihrer Funktionalität so weitsichtig geplant waren, dass sie auch heute noch nach nur wenigen Um- und Anbauten allen Ansprüchen moderner radioonkologischer Zentren entsprechen.

Prof. Strietzels wesentliches Verdienst besteht darin, dass er die Strahlentherapie stets als interdisziplinäres Fach im Umfeld anderer onkologischer Disziplinen gesehen hat und entsprechende Strukturen zur gemeinsamen onkologischen Arbeit schuf. Schon 1968 (!) gründete er an der Dresdner Klinik mit der Mund‑, Kiefer- und Gesichtschirurgie eine regelmäßige interdisziplinäre Sprechstunde, der später weitere Konzile mit anderen Kliniken folgten. In einem in der DDR erschienenen Studentenlehrbuch, für dessen Strahlentherapieteil Prof. Strietzel verantwortlich war, wurde – durch die Dresdner Erfahrungen begründet – auf die Darstellung interdisziplinärer Krebsbehandlung großer Wert gelegt und dies auch durch entsprechende Autorenschaft belegt.

Prof. Strietzel übernahm 1984 die Chefredaktion der Zeitschrift *Radiobiologia Radiotherapia*, die er bis zu deren Aufgabe im Jahr 1990 leitete.

Seine berufliche Liebe galt besonders der Brachytherapie. Für diese Behandlungsform hatte er in Rostock eine leistungsfähige Abteilung geschaffen.

Manfred Strietzel war einer der wenigen Hochschullehrer in der DDR, die sich nie der Parteidoktrin beugten. Demzufolge standen seinen Erfolgen im Beruf in dieser Zeit auch immer die verschiedensten Schwierigkeiten entgegen. Nach der politischen Wende war er in den Jahren ab 1989 an der Umgestaltung und Demokratisierung der Medizinischen Fakultät der Universität Rostock in verantwortlicher Position bis zu seiner Emeritierung (1996) beteiligt.

Auch in seiner Rostocker Zeit hielt er immer die Verbindung zu Sachsen, die sich auch in Ehrungen, so in der Verleihung der Carus-Plakette der Medizinischen Fakultät Dresden und in der Ehrenmitgliedschaft der Sächsischen Radiologischen Gesellschaft 2004, ausdrückte.

Keine Laxheit duldend, aber stets kollegial, warmherzig und den Menschen zugewandt, konnte Prof. Strietzel auf unzählige dankbare Patienten und auf eine stattliche Schülerzahl zurückblicken.

Auch in seinen letzten Jahren war er fortwährend an der Entwicklung des Fachs interessiert. Wir Jüngeren konnten in unzähligen Gesprächen stets von seinem großen Erfahrungsschatz profitieren. Erst im Frühjahr dieses Jahres hat er in einem Interview mit einem Unterzeichner über die Anfänge der Kobalttherapie 1957 in Dresden mit hellwachem Geist berichtet und die Schwierigkeiten vermittelt, mit denen die damaligen Strahlentherapeuten zu kämpfen hatten, sowie die heute unvorstellbaren Belastungen dargestellt, die sie damals ihren Patienten während der Therapiedurchführung zumuten mussten.

Seine Schüler und Nachfolger im Amt sind dankbar, Prof. Strietzel als Lehrer und Kollegen erlebt zu haben.

*Thomas Herrmann, Dresden*

*Johannes Schorcht, Dresden*

*Guido Hildebrandt, Rostock*

